# The impact of illegal waste sites on a transmission of zoonotic viruses

**DOI:** 10.1186/s12985-017-0798-1

**Published:** 2017-07-20

**Authors:** Darja Duh, Sandra Hasic, Elena Buzan

**Affiliations:** 1 Department for Medical Microbiology Maribor, Centre for Medical Microbiology, National Laboratory of Health, Environment and Food, Prvomajska 1, 2000 Maribor, Slovenia; 2 Department of Biodiversity, Faculty of Mathematics, Natural Sciences and Information Technologies, University of Primorska, Glagoljaška 8, 6000 Koper, Slovenia; 3 Institute for Biodiversity Studies, Science and Research Centre, University of Primorska, Garibaldijeva 1, 6000 Koper, Slovenia

**Keywords:** Illegal waste sites, Rodents, Lcmv, Tbev, Anthropogenic influence, Pathogen transmission

## Abstract

**Background:**

Illegal waste disposal impacts public health and causes aesthetic and environmental pollution. Waste disposed in places without permitted and controlled facilities can provide a ready source of nutrition and shelter for rodents and thus promote the spread of their ecto- and endoparasites. The presence of two distinct zoonotic viruses, lymphocytic choriomeningitis virus (LCMV) and tick-borne encephalitis virus (TBEV), was searched at illegal waste sites. The aim of this study was to determine the prevalence of infection with both viruses in rodents and to discuss the virus-rodent relations in such environments.

**Methods:**

Rodents sampled between October 2011 and April 2013 at 7 locations in the Istrian peninsula, were identified morphologically and genetically to minimize misidentification. Serological and molecular techniques were used to determine seroprevalence of infection in rodents and to detect viral RNAs. Serological testing was performed by immune fluorescence assay for detection of LCMV and TBEV specific antibodies. Real-time RT PCR was used for the detection of LCMV nucleoprotein gene and TBEV 3′ non-coding region. Data were statistically analysed using SPSS statistic v2.0.

**Results:**

Out of 82 rodent sera tested, the presence of LCMV antibodies was demonstrated in 24.93%. The highest prevalence of LCMV infection was found in commensal *Mus musculus* (47.37%), followed by 11.53%, 19.04% and 25% prevalence of infection in *A. agrarius, A. flavicolis* and *A. sylvaticus*, respectively. The highest prevalence of infection in rodents (53.33%) was found in locations with large waste sites and high anthropogenic influence. LCMV seroprevalence was significantly lower in rodents sampled from natural habitats. Viral nucleic acids were screened in 46 samples but yielded no amplicons of LCMV or TBEV. In addition, TBEV specific antibodies were not detected.

**Conclusions:**

Illegal waste sites have considerable impact on the area where they are located. Results have shown that the transmission of human pathogens can be significantly increased by the presence of waste sites. However, the pathogen must be endemic in the environment where the waste site is located. The introduction of a human pathogen as a consequence of the waste site in the area of interest could not be proven.

**Electronic supplementary material:**

The online version of this article (doi:10.1186/s12985-017-0798-1) contains supplementary material, which is available to authorized users.

## Background

One of the unpleasant by-products of urban living is municipal solid waste. A landfill is one of the major methods used for waste disposal. It is defined as the deposition of waste in a specially designated area, which consists of a pre-constructed ‘cell’ lined with an impermeable layer (man-made or natural) and with controls to minimize emissions [1].

Many urban areas cannot effectively manage their waste, which leads to continuous creation of new illegal waste sites. Worries about the effects of dumped waste on human health motivated numerous studies to investigate possible influence on human reproduction, cancer development and respiratory illnesses including asthma. Several systematic reviews have assessed the controversy over possible health effects of waste management on the public due to differences in risk communication and risk perception [1–3]. Despite that controversy, we should be more concerned about the negative impacts of illegally disposed waste upon human health and the environment. Namely, when waste such as used tires, construction debris, old appliances and furniture, as well as general household, commercial and industrial waste, is disposed in places without permitted and controlled facilities, it can provide a ready source of nutrition and shelter for rodents and consequently for their ectoparasites [4]. All of them play crucial role in the maintenance and spread of zoonotic pathogens. The waste is usually dumped on vacant lots, along utility right-of-ways, on public and private lands, and at other normally unattended locations. Because of the lack of control at illegally dumped waste sites, the threat to human health, wildlife and the environment is much higher than in controlled municipal landfills.

In the study presented here we focused on the presence of two distinct zoonotic viruses, lymphocytic choriomeningitis virus (LCMV) and tick-borne encephalitis virus (TBEV) at illegal waste sites. Although both viruses are well-known zoonotic pathogens, their ecology and epidemiology differs significantly.

LCMV is a rodent-borne prototypic member of the *Arenaviridae* family discovered in 1933 [5]. Even though it was among the first isolated human pathogenic viruses; clinically, LCMV remained less attractive for years because the infection of healthy humans with LCMV usually results in non-specific febrile illness. Nevertheless, the ability of causing a severe and permanent brain injury and dysfunction in foetuses and new-borns, and recent association of LCMV with several clusters of organ transplant transmissions makes the neurovirulent LCMV the important human pathogen [6–8]. Zoonotic exposure to LCMV occurs worldwide through aerosolized excreta or by direct rodent contact. The natural host and reservoir of LCMV is a commensal house mouse, *Mus musculus,* but the virus can be also carried by other wild rodents, pets and laboratory rodents. TBEV, on the other hand, is one of the most important tick-transmitted zoonotic pathogens, first isolated only 6 years later than LCMV. It is the aetiological agent of a potentially fatal neurological infection affecting humans in Europe and Asia [9]. Over the past 3 decades an enormous increase in tick-borne encephalitis (TBE) morbidity has been observed in Europe [10], and TBEV can now be found in regions that were previously unaffected. Extrinsic features of the environment (abiotic, biotic and human) and intrinsic biological features of the virus are described as a driving force behind the rapid spread of TBE [11]. The most important and frequent way of TBEV transmission is by tick bite, although TBEV can also be transmitted to humans with unpasteurised milk. In nature, TBEV is maintained in the zoonotic cycle involving ticks and vertebrate hosts amongst which rodents are the most important [12].

Since illegal waste sites can present optimal conditions for rodents to breed with abundant supplies of food, we were interested whether such sites with higher commensal rodent population densities also affect the occurrence of rodent-borne LCMV and TBEV. These two viruses were selected because their life cycle includes rodents; however, rodents play a distinct role for viral replication and transmission. Although 5.9% seroprevalnce of LCMV infection in wild rodents was described in Slovenia, there is a lack of data about LCMV presence in the Istrian peninsula [13]. Slovenia is endemic for TBE but the prevalence of infection with TBEV in Slovenia varies depending on the rodent and tick species and the region of trapping [14]. The incidence of TBE in Istrian Peninsula is low [15].

The aim of the study was to determine the prevalence of infection with LCMV and TBEV in *Mus* and *Apodemus* species sampled from i1llegal waste sites in the Istrian peninsula including Slovenia and Croatia and to compare the findings with the seroprevalence data obtained from natural sites. The virus-rodent relations are also discussed herein.

## Methods

### Field work

**Fig. 1:**
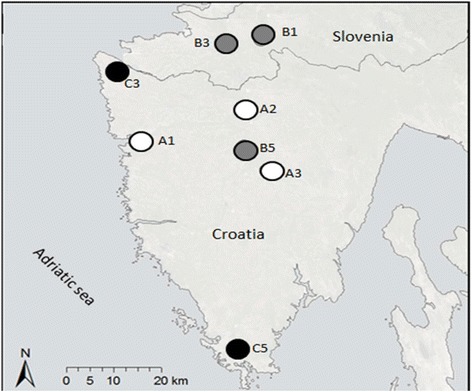
legend: Group A: Sites with little to none anthropogenic influence. Group B: Sites with medium anthropogenic influence and small waste sites. Group C: Sites with large anthropogenic influence, large waste sites and human settlements

Rodents were sampled between October 2011 and April 2013. Sampling was done in the warmer part of the year from April to November in eight locations in Istria, a peninsula in the northern Adriatic, shared by Italy, Slovenia and Croatia. The sampling sites differed significantly according to the anthropogenic impact. Tree groups of sites were identified (Fig. 1, Table 1): group A: natural habitats with low anthropogenic impact, group B: habitats with medium anthropogenic impact and small waste sites and group C: sites with high anthropogenic impact, large waste sites and human settlements.

**Table 1:** The summary data on sampling sites in Istria. Sites A1-A3 had little to none anthropogenic influence. Sites B1-B5 had medium anthropogenic influence and waste sites smaller than 0.85 ha. Sites C3 and C5 were under high anthropogenic impact, waste sites were larger than 0.85 ha and they also contained human settlements on site or nearby

Sampling site	Short description	Size (ha)
A1	Reeds near the forest	13.8
A2	Meadow near the stream	0.12
A3	Wood, road, small amount of scattered water	0.05
B1	Waste sites’ edge near the forest	0.46
B3	Meadow, small waste site, shrubs	0.71
B5	Wood, road, meadow, small waste site	0.74
C3	Large waste site, shrubs	1.72
C5	Large waste site, wood, backyard, shrubs	0.95

Traps, containing bait made of peanut butter and oatmeal or bread crumbs, were placed in the evening and examined for rodents early the next morning for five consecutive days. Animals were captured in live traps Sherman type (H.B. Sherman Traps inc. USA) of two dimensions (small Sherman traps: 50.08 × 6.35 × 22.86 cm; and large Sherman traps: 7.62 × 8.89 × 22.86 cm). In the laboratory, trained personnel euthanized the rodents using carbon dioxide. To aid identification, animals were weighed and total head-body length, tail, ear and hind foot lengths were measured. The blood, collected from the axillar artery, was centrifuged and sera were labelled and stored at −80 °C. Internal organs were labelled and stored at −80 °C until further use. Permits to work with animals and animal tissues were issued by the Ministry of Culture of the Republic of Croatia (No. 532–08–01-01/1–11-03) and the Veterinary Administration of the Republic of Slovenia (No. 34401–36/2012/9).

### Serological testing

The immune fluorescence assay (IFA) was performed with 2-well slides for LCMV provided by dr. Remi Charrel (EVA FP7 CAPACITIES Project GA No. 228292) and commercially available slides for TBEV (Anti-TBE virus IIFT, EUROIMMUN Medizinische Labordiagnostika AG, Germany). Test sera were diluted to a ratio of 1:10. To detect antibodies against LCMV, 20 μl of each dilution were loaded on the slides, incubated at 37 °C in a humidity chamber for 30 min and washed 3 times for 5 min in PBS. Anti-Mouse IgG (whole molecule-FITC, Sigma) conjugate was used at a dilution of 1:32. 20 μl of conjugate was pipetted on the slides, incubated at 37 °C in a humidity chamber for 30 min, and washed 3 times for 5 min in PBS. Slides were than dyed with Evans Blue for 5 min and washed with distilled water. Antibodies against TBEV were detected using commercial IFA slides following the instructions of the manufacturer. The exception was Anti-Mouse IgG (whole molecule-FITC, Sigma) conjugate used at dilution 1:32 instead of FITC-labelled anti-human IgG reagent provided in the kit. The fluorescence was examined with an Olympus BX51 microscope (Olympus, United Kingdom) using 40 X objective and 10 X ocular.

### Molecular identification

We used RTP DNA/RNA Virus Mini Kit (Invitek, STRATEC Biomedical AG, Germany) for the extraction of nucleic acids. In short, 1 mm^3^ large pieces of spleen were manually homogenized in 400 μl of water (molecular biology grade). The sample was afterwards processed as instructed by the manufacturer. In the final step, DNA/RNA was eluted in 200 μl of pre-warmed elution buffer.

Identification of rodents based only on morphological characteristics can lead to misinterpretations of species, especially for the juvenile individuals [16]. Therefore, we used amplification of mitochondrial cytochrome b gene for species validation.

The partial cytochrome b gene (500 base pairs) was amplified using universal primers and the polymerase chain reaction (PCR) protocol outlined in Jaarola and Searle (2002) and Jaarola et al. 2004 [17, 18]. Kappa 2G PCR multiplex kit (Kappabiosystems) was used with primers L14727- SP (5′ GACAG GAAAAATCATCGTTG’ 3) and H15497-SP (T(AG)TAATT(AG)TCNGGGTCTCC) [17, 18]. Cycling conditions consisted of an initial stage of 95 °C for 3 min followed by 30 cycles of denaturation (15 s at 95 °C), primer annealing (30 s at 60 °C) and extension (1 min at 72 °C). Final extension was performed at 72 °C for 10 min. 3 μl of DNA was used in PCR reaction for molecular identification of species. Sequencing reactions were done on ABI 3130 Genetic Analyser (Life Technologies) using BigDye Terminator Chemistry. Species were later determined using BLAST algorithm (NCBI).

A real-time RT PCR for the amplification of a 116 bp long fragment of LCMV nucleoprotein gene was performed using LCM_TM_NP1 (5′-TCATGTGGCARRATGTTGTG-3′) and LCM_TM_NP2 (5′-AAAAAGAAIAARGARATCACCCC-3′) primers, combined with a probe LCM_MAR_NP (5′-FAM-ATGATGCAATCCATAAGTGCGCAGT-DB) [19].

67 bp long fragment of TBEV 3′ non-coding region was used for the detection of specific viral RNA using F-TBE 1 (5′ GGG CGG TTC TTG TTC TCC 3′) and R-TBE 1 (5′ ACA CAT CAC CTC CTT GTC AGA CT 3′) primers combined with TBE-probe-WT (5′ FAM-TGA GCC ACC ATC ACC CAG ACA CA 3′-DB) [20].

For both reactions, we used Qiagen OneStep RT-PCR (Qiagen, Germany) reagent kit. Primers and probes were synthesized at TIB MOLBIOL (TIB MOLBIOL GmbH, Germany). The protocol of both reactions was described previously [19] [20].

Preceding the sequencing, a nested PCR was used to generate approximately 400 bp long amplicons of LCMV nucleoprotein gene. Primers used in 1^st^ round were 1817 V–LCM (5′-AIATGATGCAGTCCATGAGTGCACA-3′) and 2477C–LCM (5′TCAGGTGAAGGRTGGCCATACAT-3′), primers for the 2^nd^ round nested PCR reaction were 1902 V–LCM (5′-CCAGCCATATTTGTCCCACACTTT-3′) and 2346C–LCM (5′-AGCAGCAGGYCCRCCTCAGGT-3′). Total of 5 μl RNA was used for the RT-PCR reaction using Qiagen OneStep RT-PCR Kit (Qiagen, Germany). Cycling conditions were as follow: 50 °C - 30 min, 95 °C - 15 min, 45 cycles 95 °C - 10 s, 56 °C - 30 s, 68 °C - 20 s.

2 μl of PCR product was used in nested PCR performed under following conditions: 95 °C - 15 min, 45 cycles 95 °C - 20 s, 58 °C - 30 s, 72 °C - 40 s and 72 °C – 5 min. [19]. All sequencing reactions were done on ABI 3130 Genetic Analyser (Life Technologies) using BigDye Terminator Chemistry.

The statistical analysis was carried out using SPSS statistic v.20 (IBM Enterprise).

## Results

We screened 19 *M. musculus*, 21 *A. flavicolis*, 26 *A. agrarius*, 16 *A. sylvaticus*. Gender and stage ratios are available in Additional file 1. Most of the rodents trapped during the field sampling were not the typical host species for the viruses of interest, LCMV and TBEV, and were therefore excluded from the study.

A total of 82 rodent sera were tested with LCMV and TBEV IFA and 46 were screened for the presence of viral nucleic acids using real time RT-PCR. Serological screening of rodents for the presence of TBEV specific antibodies revealed no positive animals. Results are in concordance with the low level of TBEV endemicity in the region where rodent sampling was conducted. The level was based on TBE incidence determined according to 2000–2009 TBE incidence data [21]. It was shown recently, that detection of TBE foci can be done by testing antibodies in small mammal sera. It is less time-consuming and less expensive than molecular tools and can be an alternative approach for TBEV detection in the environment [22]. Nevertheless, molecular tools for TBEV detection were employed. They also produced negative results for all samples tested. The absence of TBEV in the region based on the serological and molecular screening was expected and confirmed [11, 21].

Specific antibodies against LCMV were detected in 20 sera (24.93% of total). The highest prevalence of LCMV infection was found in commensal *M. musculus* (47.37%). For the other wild rodent species, *A. agrarius*, *A. flavicolis* and *A. sylvaticus*, the prevalence was 11.53%, 19.04% and 25%, respectively.

Considering that all animals were trapped in a short period of time and that the animal density was low (the habitats suitable for rodents in Istria are rare and limited), the only correlation we observed was at the individual level. Results of seroprevalence indicated a possible positive correlation with weight, but statistical analysis did not confirm this trend (F = 2.565, *p* > 0.001), possibly due to the small sample size and low prevalence. LCMV prevalence was not influenced by sex (*p* > 0.001). Our results are in in agreement with data published by Tagliapiestra et al. (2009) [23] but further analysis on a larger scale should be conducted.

The seroprevalence rate was highest in the B1 location (53.33%). This site was cleaned after the first monitoring in 2011 but the seroprevalence remained high, which indicates a possibility of long-term impact on increased prevalence of LCMV even after the waste site remediation. Furthermore, the highest prevalence of LCMV was detected in habitats with high anthropogenic influence containing large waste sites and human settlements (group C: 38. 46%), and habitats with medium anthropogenic influence (group B: 36.36%), opposed to habitats with low or non anthropogenic impact (group A) which showed 17.39% prevalence of LCMV infection. Statistical analysis also confirms the trend of higher prevalence of LCMV in areas with larger waste sites and a higher anthropogenic influence (group C), opposed to smaller landfills or natural habitats (χ^2^ = 5, 256; *p* < 0,05).

## Discussion

According to our study LCMV appears to be less common in natural areas than in illegal waste sites. The impact of illegal solid waste disposal upon communities of small mammals and the potential for spreading of rodent-related diseases at the outskirts of the cities and towns has not yet been shown [24]. Cavia et al. (2009) reported that accumulated organic waste and litter provide food and harbourage for rodents. We agree that the human impact through waste disposal almost certainly changes the local species community [24, 25].

The occurrence of the commensal house mouse in the majority of illegal waste sites was proven by Buzan et al. 2013 [25]. House mouse (*M. musculus*) is a commensal species that comes to dump sites with human waste and can take the advantage of potentially rich food resources provided by such habitats. The occurrence of wild rodents was influenced by habitat and the area where waste was deposited. *A. agrarius* prefers moist river-valleys and areas with wet and dense vegetation [26] and proved to be the dominant species in natural groups of habitats and in waste sites near to water bodies [25]. The forest and dense shrub stands, where there is sporadic occurrence of fallen woody material, however, are preferred by *A. flavicollis* [27]. Its occurrence dropped evidently towards habitats with large waste sites or human settlements where it was trapped only once. The *Apodemus* species (*A. sylvaticus*) was more frequent in waste sites than other two [25]. The overall prevalence of LCMV infection among rodents in the natural environment is comparable to that reported in similar studies in Europe, where the prevalence ranged between 3% and 17% [28–31]. These results also coincide with the study from Slovenia where Bizimoska (2008) indicates 5.9% natural prevalence of LCMV in *M. musculus, A. flavicollis, Myodes glareolus, Sorex araneus* and *Glis glis* [13].

Moreover, in our study, waste sites were recognised as a possible refuge for commensal and native rodent species, which enables integration and increases possibilities of spreading rodent-related diseases. Results obtained in this study suggest that illegal waste sites can significantly contribute to the spread and circulation of the virus among their hosts. One explanation can be found in the occurrence of an edge effect. Most species were recorded in sites with medium anthropogenic impact [25] where in our case habitat was changed due to inappropriate waste disposal. The mixture of different environments at these sites and potential food resources within the landscape units around these sites allowed the species present in the region to occupy the urban ecosystem [32].

Population density also likely correlates with prevalence of LCMV infection. Despite high seroprevalence of LCMV infection in trapped rodents, real time RT-PCR for LCMV yielded no amplicons. This is in agreement with the transmission patterns of LCMV. Horizontal infections, which are acquired through direct contact with infected rodents or indirect contact with contaminated fomites and are most likely to occur on waste sites, can lead rodents to shed infectious viruses for a few weeks to a few months before virus is cleared from the animal. Only when mice are exposed in utero to LCMV, they become persistently infected and shed the virus throughout their lives [24]. Increases in population density also increase the overlap between neighbouring home ranges, the number of contacts, and conflict between rodents and thus increase the potential for virus transmission [23, 33].

Our results were consistent with Taglineapietra et al. (2009) and Laakkonen et al. (2012), who showed that the sex of the mouse does not affect LCMV prevalence. However, our results suggest that weight and sex interact and show a correlation with antibody prevalence in host populations [23, 34]. This is an indication of a horizontal transmission of LCMV by a mechanism that involves mainly males, such as infection by bite wounds inflicted during fighting [35]. This hypothesis is supported by previous reports that male mice have a greater home range than females, and their home ranges overlap more than those of more territorial females [36]. There are also studies indicating that males in sites with higher food resources become more aggressive in the group compared to those males in the group with scarce food resources [37].

Another possible explanation is that the virus might be spreading due to the deterioration of the immune response, caused by exposure to heavy metals and toxins found at illegal waste sites. Long term exposure to such factors can lower the immune protection of the host organism and thus increases the possibility of infection and transmission of the virus. However, illegal waste sites pose a risk of spreading the virus only when it is naturally present, but does not solely increase the possibility of introduction of a new virus, previously unknown to the area, as confirmed by our data. Further investigation is needed to determine the reasons for this difference.

The prevalence and transmission rates of rodent-borne viruses in host populations vary in time and space and among host-virus systems. Improving our understanding of the causes of these variations will lead to better understanding of changes in risk of disease to humans.

As a consequence, agriculture and urbanisation with the connected waste disposal activities will put humans at risk of contracting a series of rodent-related diseases [38]. Moreover, improper waste management is driving the native species to local extinctions and replacing them with invasive commensal rodents, thereby reducing biodiversity and ecosystem health [39] and bringing commensal species in contact with native rodents. Introducing commensal rodents to waste sites enables them to colonize the regions that are otherwise inhabited by native rodents, and gives them additional abilities for transmission of infectious agents from commensal rodents to native ones.

A survey of rodent ectoparasites showed that prevalence and their intensity differed significantly between natural and illegal waste sites [40]. Among all rodents.

33% of rodents were infested by ticks in illegal sites and 14% in natural sites. Despite *Ixodes ricinus* was common in illegal sites, TBEV was not detected in rodents. The increase in rodent population did not have an immediate impact on spread and increase of TBEV in the study site. Namely, rodents alone do not play a crucial role in TBEV life cycle as this is the case for LCMV. For TBEV to be present in the certain environment, complete life cycle including ticks and well as rodents and large mammals needs to be established. Understanding rodent ecology and gene flow, including movement of commensal rodents with respect to human expansion in urban landscapes, is critical for understanding the dynamics of rodent-borne pathogens and is valuable for mitigating human disease outbreaks [41].

## Conclusions

Illegal waste sites have an unavoidable and considerable impact on the area where they are located. The consequence of the illegally dumped waste on the presence and spread of human pathogens was examined. We have shown that the spread of pathogens already endemic in the environment, where the waste site is located, can be significantly increased. Namely, the seroprevalence of LCMV infection of rodents trapped at illegal waste sites was higher compared to the LCMV infection of rodents from their natural environment. The introduction of a human pathogens as a consequence of the waste site in the area of interest could however not be proven.

## Additional file

Additional file 1: Gender and stage ratio of captured animals on locations. (DOCX 13.8 kb)

## Abbreviations

IFA: Immune Fluorescence Assay
LCMV: Lymphocytic choriomeningitis virus
RNA: Ribonucleic acid
RT PCR: Reverse transcription polymerase chain reaction
TBEV: Tick-borne encephalitis virus
