# Supplementary material for: The impact of illegal waste sites on a transmission of zoonotic viruses
Source: Virol J. 2017 Jul 20;14:134. doi: 10.1186/s12985-017-0798-1 (PMC5520353; doi:10.1186/s12985-017-0798-1)
Supplement: Additional file 1: — Gender and stage ratio of captured animals on locations. (DOCX 13.8 kb) [file 12985_2017_798_MOESM1_ESM.docx]

|  | No. of captured inividuals | **Gender** | | **Stage** | |
| --- | --- | --- | --- | --- | --- |
| Locality |  | Male | Female | Adult | Juvenile |
| A1 | 3 | 2 | 1 | 3 | 0 |
| A2 | 15 | 5 | 10 | 5 | 10 |
| A3 | 5 | 2 | 3 | 4 | 1 |
| B1 | 15 | 7 | 8 | 10 | 5 |
| B3 | 12 | 10 | 2 | 8 | 4 |
| B5 | 6 | 5 | 1 | 5 | 1 |
| C3 | 13 | 8 | 5 | 10 | 3 |
| C5 | 13 | 5 | 8 | 10 | 3 |
| Total: | 82 | 44 | 38 | 55 | 27 |

Table 1: Gender and stage ratio of captured individuals at selected locations.
